# Arabic translation, cross-cultural adaptation, and testing measurement properties of the European heart failure self-care behaviour (EHFScBS-12 and EHFScBS-9)

**DOI:** 10.1371/journal.pone.0361973

**Published:** 2026-11-06

**Authors:** Lujain Adel Sallam, Nora Ghalib AlOtaibi, Homood A. Alharbi, Adnan Innab, Ahmad Hayajneh, Abeer Khamis Al.Ghafri, Osama Almogbel

**Affiliations:** 1 College of Nursing, King Saud University, Riyadh, Saudi Arabia; 2 College of Nursing, King Abdulaziz University, Jeddah, Saudi Arabia; 3 College of Nursing, Imam Abdulrahman Bin Faisal University, Dammam, Saudi Arabia; 4 King Fahad Cardiac Center, King Saud University Medical City, Riyadh, Saudi Arabia; 5 Ibri Hospital, Ministry of Health, Al Dhahira Governorate, Sultanate, Oman; 6 College of Medicine, King Saud University Medical City, Riyadh, Saudi Arabia; ISSEP Kef: Universite de Jendouba Institut Superieur du Sport et de l'Education Physique du Kef, TUNISIA

## Abstract

**Background:**

Enhanced self-care is the primary objective of many heart failure (HF) management programs. The European Heart Failure Self-care Behaviour Scale (EHFScBS), available in 12-item and 9-item versions, is widely used to assess self-care in patients with HF. However, the EHFScBS has not been translated or evaluated its measurement properties in Arabic-speaking populations with HF.

**Objective:**

The objective of this study was to translate, culturally adapt and evaluate the measurement properties of the Arabic version of EHFScBS-12 and EHFScBS-9 **followed the** COSMIN (CO**nsensus-based Standards for the selection of health Measurement Instruments) guidelines** and to compare both versions regarding structural validity, reliability, and model fit among Arabic-speaking patients with HF.

**Materials and methods:**

A cross-sectional study conducted at a governmental hospital in Riyadh used purposive sampling to recruit participants who met the predefined inclusion criteria relevant to the study objectives:137 patients with HF (mean age = 59 years; 61.3% male). The researchers evaluated the comprehensive measurement properties of the 12- and 9-item (EHFScBS) included forward-backward translation, confirmatory factor analysis (CFA), and reliability testing. In addition, convergent and divergent validity were assessed using the Self-Care Heart Failure Index version 6.2 and the Perceived Stress Scale10-Item.

**Results:**

The scale demonstrated excellent content validity as evidenced by I-CVI of .85 to 1 and S-CVI of .95, and the CFA of the 12-item scale yielded ambiguous fit indices: root mean square error of approximation (RMSEA) =0.084, (90% CI: .059−.108), comparative fit index (CFI) =0.824, and normed fit index (NFI) =0.706 which is low/suboptimal. The CFA of the 9-item scale showed good fit indices (RMSEA = 0.042, (90%CI: .000−.084), CFI = 0.969). The internal consistency was acceptable for both scales at 0.75 and 0.72, respectively. Both the versions demonstrated convergent and discriminant validity.

**Conclusions:**

The EHFScBS as unidimensional showed satisfactory measurement properties in Arabic-speaking HF patients. The 9-item one-factor has demonstrated better fit in the present sample compared to the 12-item version achieved after post-hoc modification, support its use in practice and research. However, future studies should conduct independent cross-validation in separate samples from Saudi Arabia and other Arabic-speaking countries to confirm the generalizability, and cross-cultural validity of the instrument.

## Introduction

Heart failure (HF) is one of the most debilitating diseases affecting older people and is associated with high mortality, morbidity, and healthcare expenditures [1]. HF is the end-stage of cardiovascular disease (CVDs) and has similar trends in terms of poor outcomes. Numerous studies have reported undesirable impacts of HF, as it is associated with multiple hospital readmissions, poor physical ability, reduced quality of life (QOL), and social well-being. Moreover, overwhelming family members, caregivers’ obligations, and patients’ inability to care for themselves [2,3]. Globally, it has been estimated that approximately 64.3 million people have heart failure [2]. In Saudi Arabia, the estimated HF population is 487,422 patients, with annual healthcare spending exceeding one billion American dollars, placing a substantial economic burden on healthcare systems, and increasing the risk of suboptimal quality of care [3,4].

Self-care is recognized as a fundamental principle in the management of chronic conditions, such as HF [5]. Originally conceptualized by Dorothea Orem in 1959, self-care emphasizes individuals’ responsibility to meet their own needs to maintain health, recover from illness, manage adverse effects, and assume accountability for their care [6]. Evidence demonstrates that effective self-care behaviours enable patients with HF to control symptoms, reduce mortality, and decrease hospital admissions, thereby improving their overall health outcomes [7]. Riegel et al. [8] defined self-care as a naturalistic decision-making process that supports the prevention and management of chronic illnesses, facilitates symptom perception, and guides symptom management. Self-care is a non-pharmacological approach that individuals use to maintain disease stability, health, and wellbeing [7]. In the context of HF, self-care activities include reducing salt and fluid intake, attending medical appointments, managing symptoms, and monitoring daily weight and exercise.

Reliable and valid instruments are critical for assessing and promoting effective self-care behaviours in patients with HF. Only two scales have undergone comprehensive psychometric evaluation: the European Heart Failure Self-care Behaviour Scale (EHFScBS), available in 12-item and 9-item versions [9,10] and the Self-Care of Heart Failure Index (SCHFI) [11]. Although the EHFScBS is widely used in several languages, including Spanish, Turkish, Japanese, and Chinese, and many others, a notable gap remains in the availability of a reliable, culturally adapted Arabic version to assess heart failure self-care behaviours. This gap limits researchers and clinicians in Arabic-speaking countries from accurately evaluating self-care compliance, comparing findings across populations in the literature, and implementing interventions tailored to local cultural needs. In contrast, the SCHFI has undergone rigorous psychometric evaluation in countries such as Lebanon and Yemen [12,13]. The EHFScBS was selected for this study due to its focus on patient behaviours such as limiting fluid intake, adhering to prescribed medication, and monitoring weight. Its validity, reliability, and accuracy, along with acceptable psychometric properties, have been demonstrated in clinical practice and previous research. Furthermore, the EHFScBS is considered practical because of its ease of application [14]. In contrast, the Self-Care of Heart Failure Index emphasizes the decision-making process underlying self-care behaviours [8]. As self-care behaviours are shaped by communication, culture, norms, education, and interaction, and given the global diversity of populations [15], there is a substantial need for validated research instruments that are cross-culturally applicable. Researchers and clinicians require reliable and valid tools in their respective languages and cultural contexts to conduct cross-cultural research and deliver high-quality care [16]. To the best of our knowledge, this is the first study to translate the EHFScBS into Arabic and compare both the 12- and 9-item versions using a rigorous psychometric methodology. Therefore, the objective of this study was to translate, culturally adapt and evaluate the measurement properties of the Arabic version of EHFScBS-12 and EHFScBS-9 **followed the** COSMIN (CO**nsensus-based Standards for the selection of health Measurement Instruments) guidelines** [17] and to compare both versions regarding structural validity, reliability, and model fit among Arabic-speaking patients with HF.

## Methods

### Study design

A quantitative cross-sectional methodological study was conducted to evaluate the instrument’s translation and measurement properties. The research comprised two stages: (a) translation of the EHFScBS 12-item scale into Arabic and (b) validation of the measurement properties of the EHFScBS.

### Sample and setting

The study was conducted among patients diagnosed with HF who were purposively selected during clinical visits to HF clinics at a governmental hospital in Riyadh, Saudi Arabia. Data were collected from May 19, 2025, to July 9, 2025. Eligibility screening was performed for all participants. Based on the literature for methodological studies involving translation processes, participant-to-item ratios are rules of thumb rather than formal statistical power analyses to ensure adequate statistical power for confirmatory factor analysis [18–20]. Consequently, the sample size for the 12-item scale was set at 120 patients with HF. To account for potential missing data, the sample size was increased by 10%, resulting in a final sample size of 137 participants [21].

Patients were included if they met the following criteria: (a) male or female, aged 18 years or older; and (b) inpatient or outpatient with a confirmed diagnosis of HF based on echocardiogram results (ejection fraction below or above 45%) at least three months prior to the study. Exclusion criteria were as follows: (a) diagnosis of cognitive impairments or severe psychiatric illness, such as dementia, that prevented comprehension of study instructions; (b) refusal to provide informed consent; (c) presence of life-threatening diseases; and (d) unstable clinical condition.

### Study procedure

#### The scale of the study.

The EHFScBS was originally developed with 20 items to assess self-care behaviours among patients with HF through three main phases: concept analysis and initial construction, revision of items and scoring format, and psychometric testing. Then, based on expert review, pilot testing, and item analysis, the instrument was reduced to 12 items. Initially**,** Jaarsma et al. developed the scale in Dutch in 2003 [9], followed by translations into English and Swedish. The original 12-item scale was hypothesized to encompass three dimensions: “complying with the regimen,”: Items: 1,6,9,10,11,12 “asking for help,”: Items: 3,4,5,8, and “adapting activities,”: Items: 2,7. The original study only partially confirmed the hypothesized dimensions, so the scale was used as a total score, with a reported reliability (Cronbach’s alpha) of 0.81. The EHFScBS uses a 5-point Likert scale ranging from 1 (I completely agree) to 5 (I completely disagree). The EHFScBS does not provide an established clinical cut-off for categorizing self-care behaviour; thus, total scores range from 12 to 60, with higher scores indicating poorer self-care. Following its initial development, the 12-item scale was translated into multiple languages, demonstrating applicability across diverse cultural contexts.

In 2009, Jaarsma et al. [10] conducted additional psychometric testing using item analysis, CFA, and content evaluation. The final 9-item version was derived from the original 12-item scale by removing items 2 and 7 because of weak item-total correlations, and item 11 because of weak factor loading, resulting in significant improvement over the 12-item version. The remaining items correspond to original items 1, 3, 4, 5, 6, 8, 9, 10, and 12, and the items were renumbered sequentially from 1 to 9 throughout the tables and figures to ensure consistency. The evaluation included both unidimensional and two-factor models “adherence to regimen”: Items 1, 5, 7, 8, 9 and “consulting behaviours”: Items: 2, 3, 4, 6 with scores ranging from 9 to 45, with lower scores indicating better self-care. The revised scale demonstrated adequate internal consistency (Cronbach's alpha = 0.80). Since this revision, the EHFScB-9 has been widely adopted as the short version. Evidence suggests that the scale can be used as a total score and for subscales related to consulting behaviour. However, the “adherence to regimen” subscale cannot be used independently because of its low reliability. A study by Vellone et al. [22] subsequently proposed transforming the EHFScBS-9 score to a 0–100 scale, with higher scores indicating better self-care to facilitate comparison with other studies.

Research has since examined both the 12-item and 9-item versions in various populations and languages. However, previous validation studies have reported inconsistencies in the underlying factorial structure including one, two, and three factors of the 12-item and 9-item versions [23–26]. As a result, interpretation of the EHFScBS has evolved, with ongoing debate regarding whether the instrument is best conceptualized as a multidimensional measure or as a unidimensional scale based on an overall score. Also, based on the EHFScBS's original theoretical framework, which conceptualizes self-care as encompassing adherence, consulting, and adaptive behaviours, that evaluated unidimensional and multidimensional models for both the 12-item and 9-item versions [9,10]. Therefore, both versions were examined and compared in the present study to determine which model demonstrated the preferred fit and was most appropriate for use among Arabic-speaking HF population in Saudi Arabia. Furthermore, the preferred model was selected according to predefined criteria instead of relying exclusively on the numerical superiority of individual fit indices. Moreover, to determine which version could provide a more concise and clinically feasible measure while maintaining an acceptable factor structure and psychometric performance.

## Stage 1: Translation and Cross-cultural Adaptation of the EHFScB 12-item Scale

After the scale author’s permission was obtained through email, translation into Arabic started using the forward-backward translation process as recommended by the author of the tool [20]. Two translators were recruited to conduct the step one forward translation from English into Arabic. It is generally recommended that forward translators be native speakers of the target language. In this regard, the first translator, a cardiologist, possessed expertise in the construct being measured in this study, whereas the second translator was a language expert unfamiliar with the subject matter.

The two translators worked independently and were instructed to follow the original English version closely. Each produced an Arabic version: A1 and A 2. Step two: a third bilingual translator compared the two Arabic versions to identify ambiguity and discrepancies in words, sentences, and meaning. No semantic differences were identified, only minor lexical variations. Then the research team assembled to discuss these possible differences between the two versions, then words closest in meaning to the English version has been selected. The translation process incorporated both linguistic and conceptual adaptation to achieve clarity and natural expression in Arabic. For instance, the item ‘I weigh myself every day’ was revised from ‘أقوم بوزن نفسي كل يوم’ to ‘أزن نفسي كل يوم’ following evaluation by translators and an expert panel regarding linguistic appropriateness. No substantive cultural modification of the underlying concept was necessary. The third independent translator and the research team reached an agreement based on a consensus-based approach, and they ensured that the final wording was culturally appropriate and clear for the target population (S1 File), ultimately resulting in the Arabic version A12.

Step three: backward translation, two additional translators were recruited to translate the Arabic version of the A12 into English. The back translators were native English speakers, language experts, and were unfamiliar with the constructs being measured. They were blinded to the original questionnaire and worked independently. This step produced the back-translated versions of B1 and B2. Step four: converge the two English versions into one B12 and compare them with a multi-disciplinary committee for word formatting, grammatical construction, and differences. No major differences were observed. Then the final back-translated English version was created, B12. Finally, three academic members and the research team, all fully proficient in both languages, reviewed and revised the final Arabic version to produce the version used in this study (T1). The back-translations of the EHFScBS 12-item and 9-item versions are provided in the S2 File.

### Pilot study

A pilot study was conducted to establish the face validity, readability, comprehensibility, and time required for the Arabic version of the 12-item EHFScBS. The questionnaires were distributed in a paper format in a private room during patient’s clinical visits. Purposive sampling selected 10% of the calculated sample size as recommended [20,27], and all participants included when they met the eligibility criteria of the study. Participants evaluated the scale's instructions and items using a dichotomous rating (clear or unclear) in Arabic. No statistical tests were applied and participants who found any aspect of the instructions, response format, or item comprehension unclear or noted any perceived ambiguity were asked to suggest revisions to improve clarity. Twelve native Arabic-speaking patients diagnosed with HF provided feedback from the pilot testing and indicated that the items were linguistically clear and culturally appropriate. Most participants completed the scale within five to seven minutes. Therefore, no modifications were required based on the pilot test results.

### Instruments

#### Demographic data.

The questionnaire was designed to collect personal details including age, sex, marital status, education level, employment, date of diagnosis, receiving specific education about heart failure, New York Heart Association Functional Heart Failure Class (NYHA), and ejection fraction. All questionnaires are provided as S3 File.

#### European heart failure self-care behaviour scale.

This scale, developed to assess self-care behaviours in patients with HF, consists of 12 items published in 2003. Scores ranged from 12 to 60, with lower scores indicating better self-care [9]. It was then revised into 9-items in 2009 by removing three items from the original scale, with scores ranging from 9 to 45, with lower scores indicating better self-care [10]. Each item is rated on a five-point Likert scale (1 = completely agree, 5 = completely disagree). In accordance with a previous study, to facilitate interpretation, a standardized score from 0 to 100 for the EHFScBS was applied [22]. The score was also reversed such that a higher score indicated better self-care [22]. In this study, Cronbach’s alpha of the total scale 12 and 9 are reported to be 0.75 and 0.72, respectively.

#### Perceived stress scale-10 items (PSS-10).

This Scale (PSS-10) was developed by Cohen et al.[28], and measures how stressful or overwhelming individuals feel their lives have been in the past month. Respondents answered each question on a five-point Likert scale (0 = never, 1 = almost never, 2 = sometimes, 3 = fairly often, 4 = very often). The total PSS-10 score was calculated by summing all item scores after reverse-coding positively worded items. A higher score indicated that the person felt more stressed. This scale has been translated and validated into Arabic version among Emirati students with Cronbach’s alpha was 0.67 [29]. In this study, the Arabic version of the PSS-10 was administered to patients with HF and the Cronbach’s alpha of the scale was 0.68.

#### The self-care heart failure index (SCHFI).

The Self-Care in Heart Failure Index (SCHFI) V.6, developed by Riegel et al. [11] is a 22-item instrument designed to assess self-care levels in patients with heart failure. The instrument comprises three subscales: Maintenance, Management, and Confidence. Each item is rated on a 4-point scale, and subscale scores are standardized to a range from 0 to 100, with higher scores indicating better self-care. This scale has been translated and validated among Lebanese HF patients with Cronbach’s alpha was 0.87 for the Maintenance subscale [12]. In this study, the Arabic version of the Maintenance subscale was used to assess self-care. In this study, the internal consistency of this subscale showed *(McDonald's* ω = *0.650).*

### Data collection

After initial contact and eligibility verification, trained research assistant and cardiac nurse provided the participants with a comprehensive explanation of the study’s purpose, procedures, risks, and benefits. Furthermore, the voluntary nature of participation and the unconditional right to withdraw without affecting medical care were emphasized. Following these explanations, written informed consent was obtained from all participants. Subsequently, data were collected via cardiac nurse who handled the completion procedure to avoid missing responses. For participants with literacy problem were interviewed by the nurse and were then completed. Data were collected from May 19, 2025, to July 9, 2025. The datasets used in this study are available as S4 File following appropriate procedures for anonymisation and any applicable ethical requirements.

## Stage 2: Data analysis

The data were analyzed using SPSS 31, and the factor structure was analyzed using AMOS 30. Descriptive statistics were used to summarize participants’ demographics, reporting means and standard deviations for measurement data and percentages for categorical data. Missing data and outliers were checked.

### Internal consistency

Internal consistency measures the correlation of items within the same scale and was assessed by calculating Cronbach’s α coefficient, with a value greater than 0.70, was considered as a default acceptable standard of reliability for scales used in basic research, and 0.80 as adequate/good [30,31]

### Validity

After the EHFScBS-12 was translated into Arabic and before additional measurement properties, to further determine conceptual and content equivalence of the items a panel of seven experts were used, comprising cardiologists and cardiology nurses, an academic nursing member specializing in cardiology, and PhD-level members. Content validity was evaluated by rating each item on a 4-point (Likert-type): 1 = Not relevant, 2 = Somewhat relevant, 3 = Quite relevant, 4 = Highly relevant based on wording clarity, cultural relevance, conceptual equivalence, and comprehensiveness. The scale-level content validity index (S-CVI) was calculated as the average of all item-level content validity indices (I-CVIs). Adequate content validity was defined as an S-CVI of at least 0.90 and an I-CVI of at least 0.78 [32,33]. Additionally, the Modified Kappa statistical test will be used to assess whether expert ratings exceed chance.

The construct validity was evaluated by examining convergent and discriminant validity using hypothesis testing. Convergent validity evaluates the extent to which a specific construct in the Arabic EHFScBS corresponds to an established instrument that measures the same construct. In this study, Pearson’s correlation coefficient was applied to examine the relationship between the EHFScBS and the established SCHFI v. 6.2 for self-care maintenance. we hypothesized that EHFScBS-12 and EHFScBS-9 would show a low to moderate positive correlation with SCHFI. The selection of this subscale was justified by its close content similarity to the EHFScBS-12, which assesses self-care behaviour among patients with HF [22,34]. Moreover, the Arabic translation of this subscale has been used among Lebanese HF patients.

A discriminant validity evaluation was also conducted to further strengthen the construct validity assessment. Discriminant validity was evaluated by examining how the new translation measured a distinct construct through the relationships between the EHFScBS-12 and PSS-10 items. we hypothesized that EHFScBS-12 and EHFScBS-9 would show a **weak to moderate negative** correlation with PSS-10. The selection of this scale PSS-10 items was justified by its ability to measure the effects of stress on patients’ behaviours. High levels of stress are assumed to indicate poor self-care behaviours [35]. Although the Arabic version has demonstrated acceptable measurement properties in other Arabic-speaking populations, their psychometric performance remains unestablished in Saudi patients with HF.

Structural validity was assessed using confirmatory factor analysis (CFA) in AMOS. The preferred model was selected based on several criteria, including alignment with the theoretical framework, overall model fit indices (CFI and TLI ≥ 0.90; RMSEA ≤ 0.08), changes in fit indices (ΔCFI and ΔRMSEA), **that supported the selection of the more parsimonious model (simplicity)**. Alos, because the 12-item and 9-item versions include different sets of observed variables, they were not treated as conventional nested models for chi-square difference testing. For comparisons of competing models estimated from the same data, **A**kaike **I**nformation **C**riterion (AIC) and **B**ayesian **I**nformation **C**riterion (BIC) were also considered, with lower values indicating a better balance between model fit and model complexity. Thus, model selection was based on the overall pattern of statistical fit, parsimony, and theoretical considerations rather than on any single fit index.

CFA was performed in order to evaluate the structural validity and fit on the assumed theoretical dimensions of the EHFScBS-12 as proposed by Jaarsma et al [9]. A one-factor (unidimensional) and a three-factor (multidimensional) (“asking for help”: Items: 3,4,5,8, and “adapting activities”: Items: 2,7 and “adherence to regimen”: Items: 1,6,9,10,11,12) model were tested. And the EHFScBS-9, a one-factor (unidimensional) and a two-factor (multidimensional) (“adherence to regimen”: Items 1, 5, 7, 8, 9 and “consulting behaviours” Items: 2, 3, 4, 6) model were tested [10], also Person’s correlations between factors in the multidimensional scale were provided.

Furthermore, several fit indices were used to evaluate the structural validity, including goodness of fit index (GFI), adjusted goodness of fit index (AGFI), comparative fit index (CFI), Normed Fit Index (NFI), Tucker–Lewis’s index (TLI), and root mean square error of approximation (RMSEA). The model fit was considered satisfactory when RMSEA ranged from 0.05 0.08. For GFI, AGFI, IFI, TLI, and CFI, values above 0.90 indicate acceptable fit, and ≥ 0.95 indicate good. Modification indices (MI) were considered in the 12- and 9-item versions of the scale, when theoretical and statistical rational recognized to identify a potential area for model fit improvement [36,37]

Moreover, parsimony was evaluated using the ‘negligible difference’ rule (ΔCFI ≤ 0.01; ΔRMSEA ≤ 0.015) [38]. The simpler model was selected when differences in fit were not substantial. Additionally, the AIC/BIC are statistical measures that penalize model complexity while considering model fit; lower values reflect superior model performance [37,39]

### Ethical consideration

Ethical approval for the study included recruitment, and informed consent was obtained from the Institutional Review Board (IRB) of King Saud University which covered the University hospital (KSU-HE-25-095).

## Results

### Sociodemographic and clinical variables

The sample of this study was mostly male (61.3%) with a mean age of 59.84 years. Most of the participants (65.7%) were married, 7.3% were divorced, and 10.95% were single. Regarding educational level, 43.1% of the participants had completed a university-level education. The mean duration of heart failure (HF) among the participants was 6.97 years. The majority (54.7%) were diagnosed with HF with reduced ejection fraction, 31.4% with HF with preserved ejection fraction, and 13.9% with mildly reduced ejection fraction. Most participants were classified as New York Heart Association (NYHA) Class I (47.4%) or Class II (44.5%). In terms of employment and income, 78.1% of participants were unemployed and a similar proportion (78.1%) reported satisfaction with their income. Additionally, 66.4% of the participants reported not having received any educational program about the disease, and additional details are provided in Table 1.

**Table 1:** Characteristics and Descriptive Data.

Variables	level	
**Age**	Mean ± *SD*	59.84 ± 14.74
**Gender**	Male Female	84 (61.3%) 53 (38.7%)
**Marital statues**	Married Single Divorced Widowed	90 (65.7%) 15 (10.9%) 10 (7.3%) 22 (16.1%)
**Education level**	Illiterate Primary/secondary University Other	33 (24.1%) 39 (28.5%) 59 (43.1%) 6 (4.4%)
**Employment**	Working Not working	30 (21.9%) 107 (78.1%)
**Income**	Satisfied Not satisfied	107 (78.1%) 30 (21.9%)
**Duration of the HF**	In years	6.97 ± 6.67
**Education program about HF**	Yes No	46 (33.6%) 91 (66.4%)
**NYHA Class**	Class I Class II Class III Class IV	65 (47.4%) 61 (44.5%) 10 (7.3%) 1 (.7%)
**Ejection fraction**	HFrEF below 40% HFmrEF 40%−50% HFpEF above 50%	75 (54.7%) 43 (31.4%) 19 (13.9%)

### Internal consistency

The internal consistency of the Arabic version of the EHFScBS was assessed using Cronbach's alpha. The resulting values for EHFScBS-12 and EHFScBS-9 with one-factor were (α = 0.75 and 0.72, n = 137) (Table 2), respectively, indicating acceptable internal consistency. *McDonald's omega (*ω*) has been used as* **complementary reliability indices for** EHFS**c**BS**- 12 and** EHFS**c**BS**-9,** *(McDonald's* ω **=** **0.74 and 0.72) respectively. However, the subscale** Cronbach's alpha **related the** EHFS**c**BS**-12 “adherence to regimen” showed poor internal consistency (**α = **0.52) (***McDonald's* ω **=** **0.51), and “ask for help” showed acceptable internal consistency (**α = **0.74) (***McDonald's* ω **=** **0.75), and “adapting activities” showed unacceptable internal consistency (**α = **0.31),** *McDonald's omega couldn’t be detected, items were less than three.* The subscale of EHFS**c**BS**-9 “adherence to regimen” showed poor internal consistency (**α = **0.43) (***McDonald's* ω **=** **0.45), and “ask for help” showed acceptable internal consistency (**α = **0.74) (***McDonald's* ω **=** **0.75). Overall, these findings support the evidence that the one factor model revealed acceptable internal consistency, supporting a unidimensional factor structure.**

**Table 2:** Item Analysis for The EHFScBS 12−9 Total Scale (*N* = 137).

No.	Item stems	Median (q1-q3)	12-item version		9- items version		Distribution of the item responses				
			Item-total correlation a	Cronbach's α if item deleted	Item-total correlation a	Cronbach's α if item deleted	1	2	3	4	5
1	I weigh myself every day	3 (1–4)	.265	.753	.240	.731	30.7	15.3	21.9	16.1	16.1
2	If I get SOB, I take it easy	4 (3–5)	.387	.738	__	__	4.4	8.0	24.1	26.3	37.2
3	If SOB increases, I contact my doctor or nurse	5 (3–5)	.517	.722	.518	.679	10.2	6.6	12.4	19.7	51.1
4	If leg/feet are more swollen, I contact doctor or nurse	4 (3–5)	.529	.721	.524	.679	9.5	4.4	16.1	20.4	49.6
5	If I gain 2 kilos in one week, I contact my doctor or nurse	2 (1–4)	.508	.721	.525	.675	39.4	15.3	14.6	13.1	17.5
6	I limit the number of fluids	4 (2–5)	.357	.741	.284	.721	10.2	16.1	15.3	24.8	33.6
7	I take a rest during the day	5 (3–5)	.294	.747	__	__	4.4	6.6	16.1	20.4	52.6
8	If I experience fatigue, I contact doctor or nurse	4 (3–5)	.521	.721	.548	.673	10.2	13.9	19.0	19.7	37.2
9	I eat a low salt diet	4 (3–5)	.512	.723	.509	.682	10.2	6.6	21.2	24.1	38.0
10	I take my medication as prescribed	5 (5–5)	.200	.754	.158	.732	2.9	1.5	3.6	10.2	81.8
11	I get a flu shot every year	3 (1–5)	.296	.753	__	__	35.0	12.4	11.7	8.8	32.1
12	I exercise regularly	3 (2–4)	.301	.748	.291	.722	23.4	17.5	21.9	16.1	21.2
Total scale		64.5 (51–77)	0.75**b**		0.72**b**						

^a^Corrected for overlaps.

^b^Cronbach's alpha for the EHFScBS total scale.

^c^SOB, shortness of breath.

### Face and content validity

The face and content validity were assessed by seven experts in the field, and the CVI results showed an I-CVI ranged from .85 to 1, indicating that all items are relevant, and S-CVI of .95, indicating excellent content validity of the translated scale. Thus, all items were retained on the scale. Additionally, the modified kappa coefficient for each item ranged between .84 to 1, indicating excellent agreement among experts beyond chance, the table is provided as S5 File.

### Construct validity

#### Convergent and discriminant validity.

Convergent and discriminant validity were assessed for both the 12-item and 9-item versions of the EHFScBS (unidimensional) by using established questionnaires. As hypothesized, the findings provide evidence of convergent validity with a low to moderate positive correlation of (*r* ≈ 0.32 and 0.30, respectively) (*P* < 0.001) with the SCHFI V 6.2 maintenance subscale. And as expected, negative correlations were observed between the 12-item and 9-item versions of the PSS-10 (*r* = −.278 and −.234, respectively). These findings support the hypothesis that the scale demonstrates adequate discriminant validity.

#### Item analysis.

No missing responses were observed for any item. Item analysis of the 12-item EHFScBS total scale indicated that most items exhibited skewness values that ranged from negative to near-normal. In contrast, items 1, 11, and 12 displayed near-symmetrical distributions. The item-total correlations ranged from 0.2 to 0.529 for EHFScBS-12 and from 0.158 to 0.548 for EHFScBS-9 (Table 2). Most items demonstrated a satisfactory item-total correlation of ≥ 0.3. Only one item (10) had a weak item-total correlation of 0.2 or lower in both versions. However, the deletion of this item did not improve the internal consistency of the scale. Thus, it was decided to retain item 10 on the scale.

#### Confirmatory factor analysis.

The CFA was used to assess the structural validity of a measure. The CFA of the EHFScBS-12 with three factors showed poor model of fit RMSEA = .090 (poor) with (90% CI: .066−.114), GFI = .885 (acceptable), AGFI = .825 (marginal), CFI = .805 (poor), TLI = .748 (poor), and NFI = .697 (poor), suggesting that the hypothesized model did not adequately fit the data (S6 Fig). Correlations among the three factors were examined. Adherence was strongly correlated with Asking (r = .727) and Adapting (r = .906). Asking and Adapting were also strongly correlated (r = .785).

Followed by CFA of the EHFScBS-12 with one factor model, the model exhibited mixed fit indices: RMSEA = .094 (poor) with (90% CI:| .071−.117), GFI = .816 (marginal), AGFI = .816 (marginal), CFI = .774 (poor), TLI = .724 (poor) and NFI = .663 (poor), suggesting that model did not adequately fit the data. In contrast, the final modified model that included post-hoc correlated residual showed RMSEA = .084 (marginal) with (90% CI: .059−.108), GFI = .887 (marginal), AGFI = 0.833 (poor), CFI = .824 (marginal), TLI = .780 (poor), and NFI = .706 (poor), suggesting that the overall model fit was suboptimal (Fig 1). In terms of parsimony, the three-factor model for the 12-item version did not show improved fit over the one-factor model (ΔCFI = −0.019; ΔRMSEA = +0.006), as these values did not exceed the threshold for a negligible difference. And for the 12-item version, the one-factor model showed lower AIC (153.572 vs. 160.889) and BIC (226.572 vs. 239.728) than the three-factor model.

**Fig 1:**
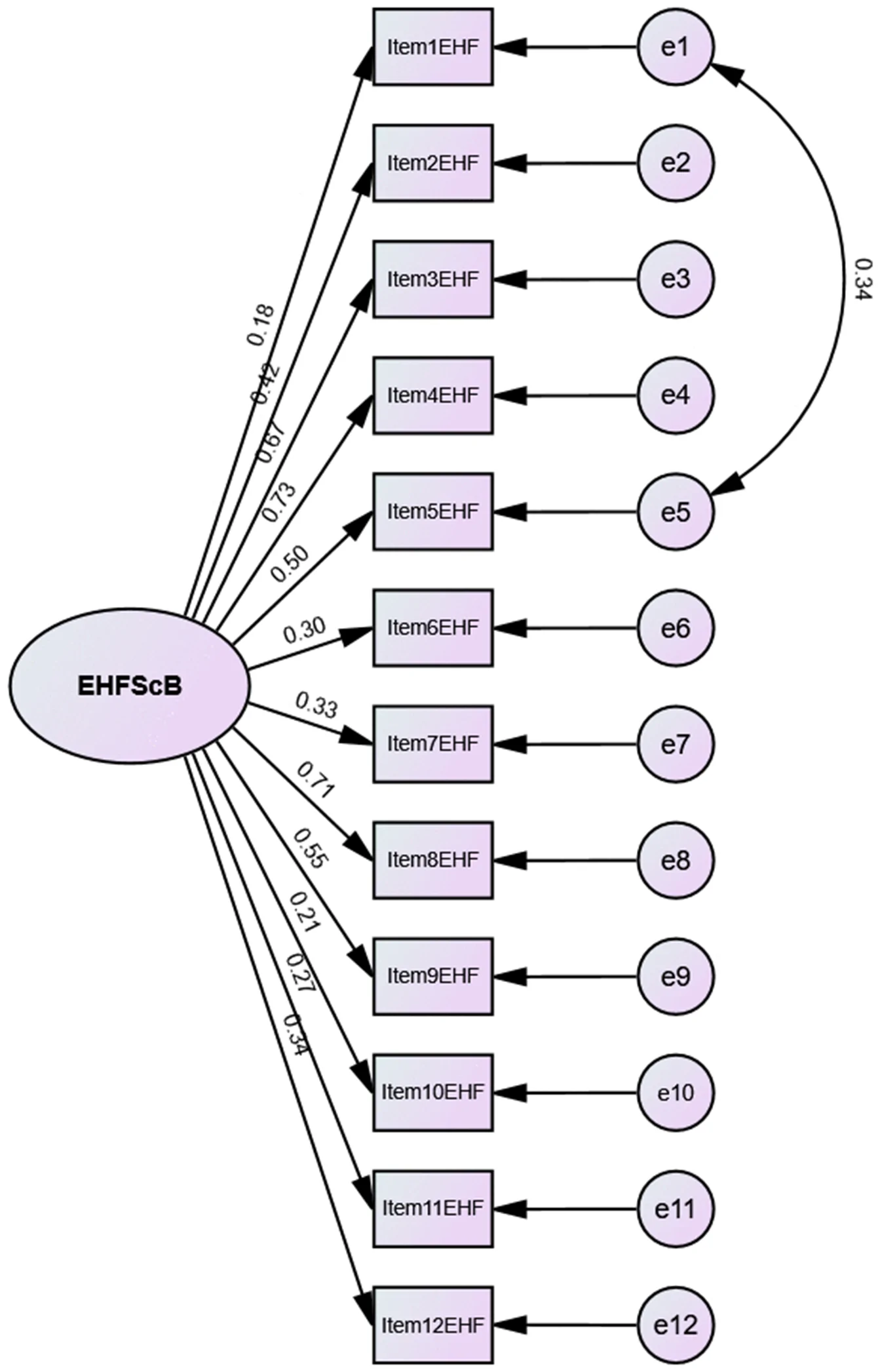
Confirmatory Factor Analysis of the EHFScBS-12 One Factor Model Arabic Version (n = 137).

The CFA of the EHFScBS-9 with two factors model showed acceptable model of fit, RMSEA = .068 (acceptable) with (90% CI: .026−.104), GFI = .938 (acceptable), AGFI = .892 (marginal), CFI = .920 (acceptable), TLI = .889 (marginal), and NFI = .824 (marginal), suggesting that the overall model fit was acceptable, although some of the indices were below the recommended threshold (S7 Fig). Correlations between the two factors of Adherence and Asking were examined. A strong correlation was identified (r = .729). And the CFA was performed on the 9-item version of the EHFScBS with one factor and three items removed (items 2, 7, and 11). The model exhibited mixed fit, as indicated by RMSEA = .077 with (90% CI: .040−.111) (acceptable), GFI = .926 (acceptable), AGFI = .876 (marginal), CFI = .895 (marginal), TLI = .859 (marginal), and NFI = .798 (poor), suggesting that the model showed marginal fit. In contrast, the final modified model that included post-hoc correlated residual yielded RMSEA = .042 with (90% CI: .000−.084) (good), GFI = .949 (acceptable), AGFI = .912 (good), CFI = .969 (good), TLI = .958 (good), and NFI = .866 (marginal), suggesting that the model exhibited good fit among the majority of the indices (Fig 2). Also, the two-factor model demonstrated poorer fit than the one-factor model (ΔCFI = −0.049; ΔRMSEA = +0.026), indicating that the one-factor model was more parsimonious. Furthermore, the one-factor model had lower AIC (70.281 vs. 80.427) and BIC (125.761 vs. 135.907) values than the two-factor model.

**Fig 2:**
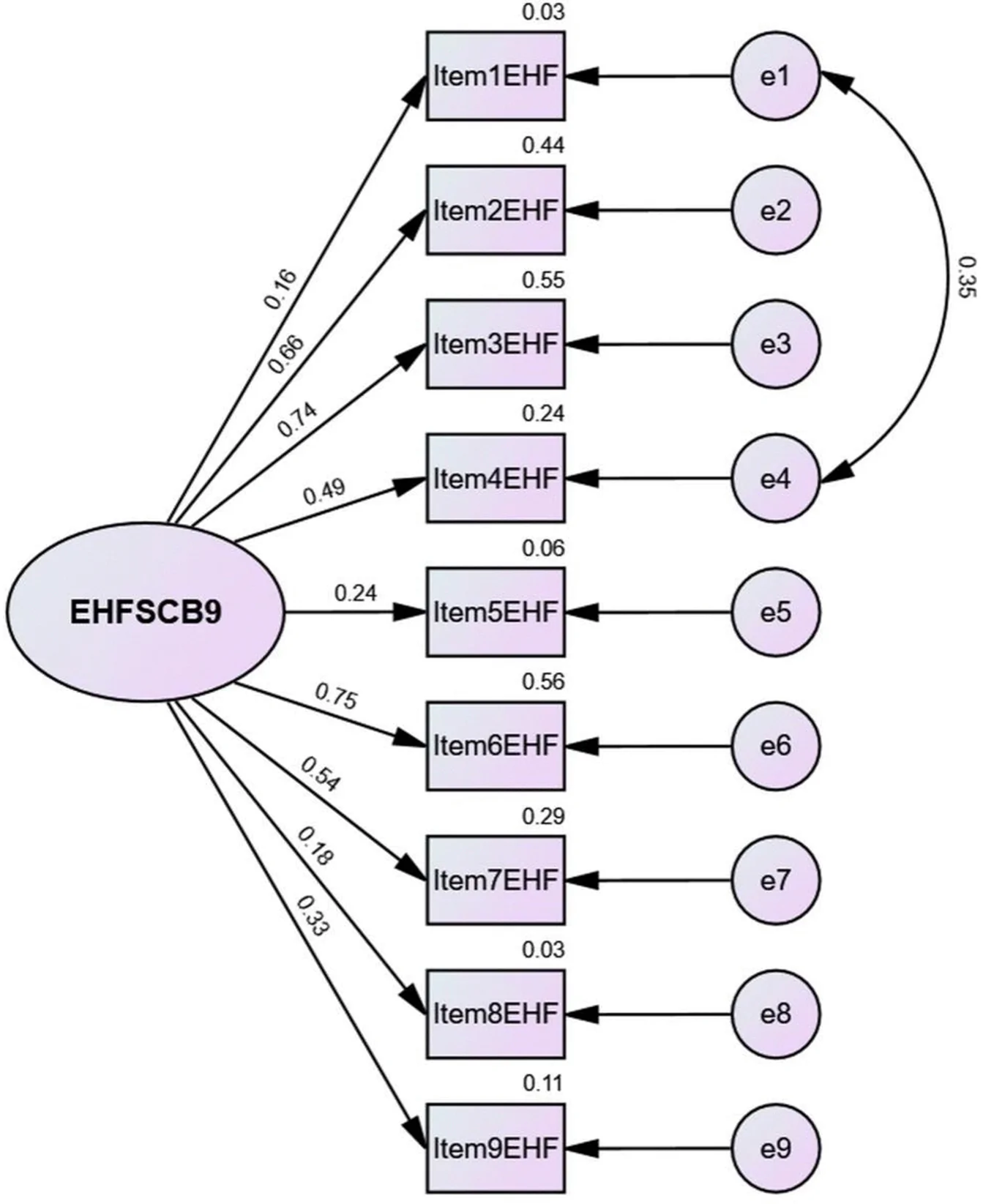
Confirmatory Factor Analysis of the EHFScBS-9 One Factor Model Arabic Version (n = 137).

Analysis of modification indices (MIs) for the 12-item and 9-item scales using a one-factor model indicated the largest MIs and suggested correlate residual between items 1 and 5 in the 12-item version, and between items 1 and 4 in the 9-item version. Specifically, item 1 (“I weigh myself every day”) and item 5, as well as item 4 (“If I gain 2 kilos in one week, I contact my doctor or nurse”), demonstrated MIs of 14.256 and 15.259, respectively.

Overall, the CFA of the 12-item version produced ambiguous results for both the one-factor and three-factor models. In contrast, the 9-item unidimensional Arabic version demonstrates better model fit than the 12-item with one-factor version, thereby supporting enhanced structural validity (Table 3).

**Table 3:** Model Fit Summary (N = 137).

Models	χ2	df	χ2/df	p-value	GFI	AGFI	CFI	TLI	RMSEA (90%CI)	NFI
12-item one-factor	103.572	53	1.954	0.000	0.887	0.833	0.824	0.780	0.084 (.059−.108)	0.706
Three-factor	106.889	51	2.096	0.000	0.885	0.825	0.805	0.748	0.090 (.066−.114)	0.697
9-item one-factor	32.281	26	1.242	0.184	0.949	0.912	0.969	0.958	0.042 (.000−.084)	0.866
Two-factor	42.427	26	1.632	.022	0.938	0.892	0.920	0.889	0.068 (.026−.104)	0.824

AGFI = Adjusted Goodness of Fit Index; CFI, comparative fit index; GFI, Goodness of Fit Index; NFI = Normed Fit Index; RMSEA, root-mean-square error of approximation; TLI, Tucker–Lewis’s index.

In terms of factor loading, for the 12-item one-factor model ranged from .18 to .73, while those for the three-factor model ranged from .27 to .74. For the 9-item one-factor model, loadings ranged from .16 to .74, and for the two-factor model, they ranged from .25 to .75 (Table 4).

**Table 4:** Factor loading for the four measurement models on the EHFScBS Based on the Confirmatory Factor Analysis (n = 137).

Original items	One factor model, 12 items	Three factor model, 12 items			New items, nine-version	One factor model, nine items	Two factor model, nine items	
		Factor I	Factor II	Factor III			Factor I	Factor II
**1**	.18	.27	–	–	1	.16	.26	–
**6**	.30	.43	–	–	5	.24	.35	–
**9**	.55	.64	–	–	7	.54	.72	–
**10**	.21	.29	–	–	8	.18	.26	–
**11**	.27	.35	–	–	Removed	–	–	–
**12**	.34	.34	–	–	9	.33	.33	–
**3**	.66	–	.68	–	2	.66	–	.67
**4**	.73	–	.74	–	3	.74	–	.74
**5**	.50	–	.49	–	4	.49	–	.49
**8**	.71	–	.73	–	6	.75	–	.75
**2**	.42	–	–	.45	Removed	–	–	–
**7**	.32	–	–	.40	Removed	–	–	–

#### The Floor and ceiling effects.

Table 5 shows the results for the floor and ceiling effects for both versions of EHFScBS 12 and 9 total scale. The floor effect was 0% and the ceiling effect was 1.5 in the 9-item version. For the 12-item version, both floor and ceiling effects were 0%. All values were well below the recommended 15%, suggesting that there were no floor or ceiling effects in the two versions of the scale [40]

**Table 5:** Floor and Ceiling effects of the European Heart Failure Self-care Behaviour Scales 12 and 9 (n = 137).

Total Scale	Mean ± SD	Floor effect	Ceiling effect
**EHFScBS-12**	63.54 ± 1.49	(0%)	(0%)
**EHFScBS-9**	62.93 ± 1.60	(0%)	2(1.5%)

Abbreviations: SD, standard deviation. All scale scores were standardised to 0–100 points, with higher scores indicating better self-care; Data are presented as n (%); Floor and ceiling effects, refer to the percentages of patients who obtained the minimum and maximum possible scores, respectively.

## Discussion

The objective of this study was to translate, culturally adapt and evaluate the measurement properties of the Arabic version of EHFScBS-12 and EHFScBS-9 **followed the** COSMIN (CO**nsensus-based Standards for the selection of health Measurement Instruments) guidelines** [17] and to compare both versions regarding structural validity, reliability, and model fit among Arabic-speaking patients with HF. The translation process followed the established international guidelines [20]. A valid self-care questionnaire is essential for assessing behavioural changes. To the best of our knowledge, this is the first study to translate the EHFScBS into Arabic and compare both the 12- and 9-item versions using a rigorous psychometric methodology. The findings of the scale translation indicated that semantic, idiomatic, experiential, and cultural equivalence was achieved with the back-translation of the A-EHFScBS and the involvement of a panel of seven bilingual healthcare professionals. The content validity of the present study was high and confirmed that all items of the scale were relevant, I-CVI ranged from .85 to 1 and the S-CVI was .95 indicated excellent content validity of the translated scale. Moreover, the findings of this study suggested that both the 12- and 9-item EHFScBS are better to use as a unidimensional measure of self-care, suggesting that these items assess a single underlying construct in patients with HF.

The confirmatory factor analysis initially supported the original study by Jaarsma et al. [10] for both scale versions with satisfactory fit indices. In this study, The CFA results for the 12- and 9-item EHFScBS indicated that the 12-item one- and three-factor models yielded ambiguous outcomes, whereas the 9-item one- and two-factor models of self-care demonstrated a superior fit. Factor correlation analysis showed that the 12-item model exhibited very strong correlations (r = .72 to .90), indicating substantial overlap among dimensions. This raises concerns about discriminant validity at the dimensional level, and this is another reason to recommend using the scale as unidimensional. However, in the original study of the three-factor model, the factor “adapting activities” showed weak correlations with the other two factors, “asking for help” (r = 0.37) and “adherence with the regimen’ (r = 0.20) [9]; hence, the items 2 and 7 of “adapting activities” factor have been removed. In the present study, the observed differences may result from a combination of cultural interpretations of scale items and sample-specific factors, rather than the scale's inherent properties [41]. Also, participants were recruited from a single region and hospital, which may have limited the range of self-care behaviours assessed.

In the 9-item model, the two factors were also strongly correlated (r = .72). Nevertheless, the two-factor 9-item model showed inadequate fit (RMSEA = .077), suggesting the remaining dimensions were not sufficiently distinct in this sample. Although, in the original author's study of the 9-item scale, the correlation between the two factors was strong (r = .83) [10], the reliability of the “adherence to regime” factor demonstrated low internal consistency. Therefore, the author suggested to use the 9-item scale as a total score as well the subscale of “ask for help” factor. Notably, in the present study the 9-item scale with one factor exhibited a good fit to the data RMSEA = .042, this fit was achieved after post-hoc modification. This indicates the items in this model measure a single underlying construct in patients with HF. These results align with the findings of Jaarsma and colleagues, the tool's developers [10].

Furthermore, model selection followed parsimony and comparative model fit, using pre-specified thresholds for meaningful differences the 9-item version, the one-factor model yielded lower AIC and BIC values than their multidimensional counterparts. Therefore, the one-factor models offered a more parsimonious presentation without compromising model fit or theoretical interpretability. Overall, the 9-item one-factor model, in particular, demonstrated the most preferable combination of fit, parsimony, and theoretical coherence. Furthermore, the 9-item version offers advantages such as conciseness, user-friendly as it reduced individual burden, and shorter completion time, making it more practical for patients and routine clinical assessment. This concise format may also facilitate implementation in busy clinical settings and improve the efficiency of screening and assessment. This finding aligns with several studies in literature explicitly noted that 9-items version easy to administer and more feasible and well-fitting factor structure [10, 22, 25, 42].

Analysis of modification indices (MIs) for the 12-item and 9-item scales using a one-factor model indicated the largest MIs and suggested correlate residual between items 1 and 5 in the 12-item version, and between items 1 and 4 in the 9-item version. These modifications were identified post-hoc and were theoretically justified [8]. Daily symptom monitoring (Item 1) is conceptually linked to the subsequent behavioural response to specific warning signs (Item 5, Item 4). Additionally, HF clinical guidelines emphasize daily body weight monitoring and weight changes, instructing patients to incorporate these behaviors into their self-care routines. This approach is further supported by the understanding that even though these items are hypothesized to represent different dimensions of self-care, their high modification indices (MIs) indicate a logical order from monitoring to action. This order provides clear data to detect early signs of fluid retention. In turn, this aligns with the theoretical framework, which conceptualizes self-care as encompassing both maintenance and management behaviours [8]. Although the covariances among these error terms in the 12-item version reduced the RMSEA from .094 to .084, the overall model fit indices indicated an inadequate fit. While, in the 9-item version, items were allowed to correlate led to improve the model fit RMSEA from 0.077 to 0.042 indicated good fit.

Following the confirmatory factor analyses and a comprehensive evaluation of measurement properties and item content, the 9-item version (unidimensional) was found to be internally consistent and has better construct validity than the EHFScBS-12 for measuring self-care behaviour in individuals with HF. Therefore, the 9-item scale as one factor was considered. These findings are consistent with those of previous studies that addressed the measurement properties of the EHFScBS 9-item Jaarsma et al. [10], and Uchmanowicz et al. [43] reported only one factor (unidimension). Also, previous research conducted for Hebrew, Kazakh, and Persian versions [10,25,26,44]. Suggested that 9-item scale better to use as a unidimensional. In contrast, in related studies, Baydemir et al. identified four factors [45], Vellone et al. and Köberich et al. identified three factors [22,46], Lee et al., Østergaard et al. and Gal et al. identified two factors [14,26,47] for the EHFScBS-9 scale, which different from the present study's findings.

Although the factor loadings were below 0.30, which is slightly less than the generally accepted threshold, these items were retained to preserve the integrity of the self-care construct. Before expert validation, the Arabic translations were considered meaningful and necessary for evaluating self-care behaviours in HF patients. The decision to delete or retain an item was determined by the content validity experts, the items theoretical importance. Accordingly, the items with weaker loadings could be retained [48]. The factor loading for item 1 (daily body weight) was below 0.30 in both scale 12 and 9 Arabic versions. In contrast to the findings of Jaarsma et al. [10] item 1 demonstrated a strong loading. In the present study, the low loading observed for item 1 may be due to cultural factors; in some Arab cultures, individuals may not routinely weigh themselves daily, viewing the practice with distrust or as unnecessary. Additionally, practical barriers exist, such as limited access to scales for those with mobility limitations, or reliance on family members for assistance and inadequate patient education about the importance of monitoring disease symptoms [49]. The low factor loading of item 1 may slightly compromise the measurement of the intended factor, raising concerns about structural validity. Despite the low loading of item 1, it was retained in the scale because its removal would not enhance reliability. Furthermore, the theoretical significance of item 1 for HF-specific self-care behaviours remains a priority. Moreover, item 10 (adherence to prescribed medication; 12-item version) and item 8 (adherence to prescribed medication; 9-item version) were also below 0.30. The low loading identified for the item related to medication adherence was consistent with the findings reported in several studies [10,26,42]. In Arab culture, self-care behaviours such as medication adherence are shaped by external factors, including family support and educational background [49]. In the present study, most participants resided with a spouse or family members; however, the majority lacked adequate education about their disease. Insufficient education may hinder patients’ understanding of the significance of adhering to prescribed medication regimens. This relationship is supported by a systematic review, which found that insufficient education about their diagnosis and difficulty following instructions correctly contribute to poor health behaviours, including medication adherence [50]. Moreover, this result may be attributed to the limited variability in the response categories, which is likely due to the homogeneity of the sample, as all patients were recruited from a single healthcare setting.

Furthermore, the loading of item 11 (I get a flu jab every year; 12-item version) was low relative to the recommended threshold, indicating it was less representative of the latent construct in this sample. In Saudi Arabia, receiving flu vaccine is driven primarily by perceived risk, limited knowledge and awareness, physicians’ requests, and concerns about vaccine safety and side effects, rather than routine health practices [51] Many participants in this cultural context do not regard receiving a flu vaccination as self-care, particularly because it is an annual practice rather than a routine part of HF management. This finding aligns with the results reported by the scale's original developer, who subsequently removed this item in the revised version of the scale [10].

Moreover, item 5 (I limit the amount of fluids I drink [not more than 1.5–2 liters per day]; 9-item version) demonstrated low loading. Prior Saudi studies have reported several challenges related to cultural and environmental factors, health literacy, and disease-specific knowledge among patients, which can negatively affect adherence to self-care required for effective HF management [52,53]. Fluid restriction is generally not considered a healthy behaviour and it may conflict with their cultural identity. In hot climates, adequate hydration and fluid intake are promoted as healthy habits among the Saudi population. Additionally, cultural practices that emphasize hospitality and beverage consumption in social contexts may hinder the implementation of fluid restriction. Further analysis of the low loading indicated that participants lacked sufficient knowledge regarding the role of fluid restriction in HF management. Consequently, this behaviour was not commonly practiced as part of disease self-care.

This study assessed the floor and ceiling effects for the standardized total scores of both the 12- and 9-item versions of the EHFScBS, addressing a gap in reporting observed in several previous validation studies. The results indicated minimal clustering of responses at the extremes of the scale. The 12-item version showed floor and ceiling effects were each 0%. Whereas the 9-item version showed floor and ceiling effects of 0% and 1.5% with two participants, respectively. These values are well below the commonly accepted 15% threshold for psychometric assessments, indicating that neither version presents a significant floor or ceiling effect. These findings were similar to those of the Italian study, which reported a floor effect of 0% and a ceiling effect of 1% [22] While in Hebrew study, which reported floor and ceiling effects of 2% and 1% for the 9-item version, respectively [26]. Consequently, the A-EHFScBS can capture a wide spectrum of self-care behaviours among patients with heart failure and effectively differentiate between varying levels of self-care. The absence of extreme score clustering further supports the instrument's sensitivity and provides evidence of its construct validity. Additionally, the findings indicated that both versions possessed adequate discriminative validity.

Moreover, convergent validity was demonstrated between both the 12-item and 9-item versions and the SCHFI V 6.2 maintenance subscale (*r* = 0.32 and 0.30, respectively; *P* < 0.001). Scales demonstrated a positive correlation, indicating that they assess related aspects of HF self-care. These results are consistent with those of the previous studies [10,14,26,42]. However, the magnitude of the correlation was low to moderate. This result may be attributed to differences in the content and conceptual focus of the measures. Specifically, the SCHFI partially reflects the process of decision-making involved in patients’ HF self-care [8], whereas the EHFScBS emphasizes the performance of self-care behaviours. Therefore, complete overlap between the instruments is not anticipated. Moreover, the relatively low internal consistency of the SCHFI Maintenance subscale in the present sample (ω = 0.650) is a limitation. Measurement error at this reliability level may have reduced the observed association with the EHFScBS, therefore, interpret the convergent validity findings with caution. Conversely, negative correlations were found between the 12-item and 9-item versions and the PSS-10 (*r* = −.278 and −.234, respectively), supporting adequate discriminant validity.

Additionally, The Arabic versions of the SCHFI and PSS-10, used as comparator instruments, have undergone psychometric evaluation in other Arabic-speaking populations; however, their measurement properties have not been specifically established among Saudi patients with HF. While previous studies support the reliability and validity of these instruments in Arabic-speaking contexts, cultural, linguistic, and clinical differences may affect their psychometric performance across populations [12,29]. Thus, interpret the convergent and discriminant validity findings in the present study with caution, as they may not fully reflect the performance of these comparator instruments in the Saudi HF population.

The internal consistency of the 12-item and 9-item Arabic versions was assessed using Cronbach's alpha. The Cronbach's alpha values for the one-factor 12-item and 9-item scales were 0.75 and 0.72, respectively, which are acceptable, as values of 0.70 or higher indicate sufficient internal consistency [30,54]. However, certain subscales showed relatively lower internal consistency in the present sample, the findings suggest that interpreting the subscales separately does not provide sufficiently precise measurements. Consequently, subscale results should be interpreted with caution. Hence, these findings confirm that the unidimensional scale demonstrated adequate internal consistency and is recommended for use as a total score. These results are consistent with those reported for the original tool developer of EHFScBS [10]. Comparable internal consistency values of the total score, ranging from approximately 0.70 to 0.80 for the 9-item and 12-item versions, have been observed in other language adaptations, including Brazilian (α = 0.70) [55], Germany (α = 0.71) [46], Japanese (α = 0.71) [24], Hebrew (α = 0.78) [26], Persian (α = .72) [23], and Slovak (α = .77) versions [42]. The current Arabic translation demonstrated slightly higher internal consistency than the United Kingdom version (α = 0.68) [56] However, the Kazakh and Korean versions exhibited even higher internal consistency (α = 0.82 and α = 0.84, respectively) [23–26].

Having a validated Arabic version enables participation from diverse populations in research, enhances cross-cultural comparability, and helps healthcare providers better identify gaps in self-care. Also, it will help in designing culturally appropriate interventions and fostering patient-centered care within the Arabic-speaking context.

### Strengths and limitations

A notable strength of the present study is that it employed a standardized translation approach with rigorous linguistic validation procedures and complemented these with comprehensive statistical analyses to assess the scale's reliability and validity. Moreover, the present study has assessed floor and ceiling effects, which were lacking in several validation studies in the literature. However, several limitations have been identified. The cross-sectional design limits conclusions about responsiveness and predictive validity and prevents assessment of reliability aspects that require repeated measurements over time, such as stability. Longitudinal studies are necessary to evaluate these properties. Also, a mixed-administration method (self-report vs. interview) may increase vulnerability to biases, including social desirability. Additional limitations include single-center recruitment, purposive sampling bias, and a limited sample size (N = 137) is borderline for complex confirmatory factor analysis. A small sample size may compromise the stability of parameter estimates and reduce the statistical power to differentiate between competing models. Future research should validate the factor structure using a larger, independent sample. Also, A limitation of this study is that the post-hoc were made to improve the CFA model fit, this may have capitalized on sample-specific characteristics, especially given the modest sample size. Ideally, the modified structure should be evaluated and cross-validated in an independent sample to establish its robustness and generalizability. However, independent cross-validation was not feasible in this study. Consequently, interpret the findings with caution, and future research with independent, larger samples is needed to confirm the stability of the modified structure. Another limitation involves the reliability of the SCHFI Maintenance subscale, which served as the comparator for assessing convergent validity. In this sample, the subscale demonstrated relatively low internal consistency (McDonald’s ω = 0.650). Consequently, interpret the convergent validity findings with caution. Furthermore, test-retest reliability and responsiveness were not assessed.

Another limitation is that the Arabic versions of the SCHFI and PSS-10, used as comparator instruments, have undergone psychometric evaluation primarily in Arabic-speaking populations outside of Saudi Arabia. Consequently, interpret the convergent and discriminant validity findings in this study in light of the limited validation evidence specific to Saudi patients with HF. Additionally, the scale was validated using standardized Arabic among the Saudi population; however, cognitive interviewing was not conducted with participants from other Arab countries. Therefore, caution should be exercised when generalizing these findings to other Arab populations.

## Conclusion

This study found that the Arabic translation of the EHFScBS demonstrated good validity and reliability as a unidimensional instrument in a sample of HF patients from Saudi Arabia, making it feasible for use in clinical and research settings to evaluate self-care behaviours in patients with HF. The Arabic 9-item (unidimensional) EHFScBS was shown to be internally consistent and valid compared to the 12-item version. The low reliability of some subscales suggested that these dimensions should not be considered independently reliable measures. Future studies should further validate the Arabic 9-item scale among Saudi and other Arabic-speaking HF populations.

## Supporting information

S1 File Forward and backward translation and cultural adaptation procedure. (DOCX)

S2 File The back-translation of the EHFScBS 12-item and 9-item. (DOCX)

S3 File Questionnaires. (PDF)

S4 File Dataset. (XLSX)

S5 File I-CVI_S-CVI_Modified_Kappa_12items_7 experts. (XLSX)

S1 Fig Confirmatory Factor Analysis of the EHFScBS-12 Three Factor Model Arabic Version (n = 137). (DOCX)

S2 Fig Confirmatory Factor Analysis of the EHFScBS-9 Two Factor Model Arabic Version (n = 137). (DOCX)
