# Supplementary material for: Arabic translation, cross-cultural adaptation, and testing measurement properties of the European heart failure self-care behaviour (EHFScBS-12 and EHFScBS-9)
Source: PLoS One. 2026 Nov 6;21(11):e0361973. doi: 10.1371/journal.pone.0361973 (PMC13742870; doi:10.1371/journal.pone.0361973)
Supplement: Supplementary file 1 [file pone.0361973.s003.pdf]

Sociodemographic Questionnaire

**Sociodemographic Characteristics and Clinical Characteristics**	**الخصائص الاجتماعية الديموغرافية والخصائص السريرية**
**Age**: ……….. years	**العمر**..........سنة
**Sex**:<br>o Male<br>o Female	**الجنس**<br>o ذكر<br>o أنثى
**Marital Status**:<br>o Married<br>o Single<br>o Divorced<br>o Widowed	**الحالة الاجتماعية:**<br>o متزوج/ة<br>o أعزب/عزباء<br>o مطلق/ة<br>o أرمل
**Educational Level**:<br>o Illiterate<br>o Primary/Intermediate/Secondary Education<br>o University Education or higher<br>o Other	**المستوى التعليمي:**<br>o أمي<br>o تعليم ابتدائي/متوسط/ثانوي<br>o جامعي<br>o غيرذلك
**Employment**:<br>o Working<br>o Not Working	**الحالة الوظيفية:**<br>o يعمل/تعمل<br>o لا يعمل/ لا تعمل
**Income (as perceived by the patient)**:<br>o Satisfactory<br>o Unsatisfactory	**الدخل المادي:**<br>o مرضي<br>o غير مرضي
**Date of diagnosis**:<br>……………….. in months/years	**تاريخ التشخيص:**<br>....................... شهر/سنة
**Receiving Specific Education About the Disease**:<br>o Yes<br>o No	**تلقي برنامج تعليمي محدد حول المرض:**<br>o نعم<br>o لا
**New York Heart Association Functional Heart Failure Class**:<br>o (I)<br>o (II)<br>o (III)<br>o (IV)	**تصنيف فشل القلب الوظيفي لجمعية القلب في نيويورك:**<br>o (I)<br>o (II)<br>o (III)<br>o (IV)
**Ejection Fraction**<br>o HFrEF below 40%<br>o HFmrEF 40-50<br>o HEpEF above 50%	o HFrEF أقل 40%<br>o HFmrEF 40%-50%<br>o HEpEF أعلى 50%

The European Heart Failure Self-care Behaviour Scale

This scale contains statements about self-care for heart failure. Please answer each statement by circling the number that you think best applies to you. Note that the response options form a scale ranging from "Completely agree" (1) to "Do not agree at all" (5). Even if you are unsure about a particular statement, circle the number that feels most accurate for you.

	**I completely agree**				**I don't agree at all**
1. I weigh myself every day	**1**	**2**	**3**	**4**	**5**
2. If I feel short of breath, I take it easy	**1**	**2**	**3**	**4**	**5**
3. If my shortness of breath increases, I contact my doctor or nurse	**1**	**2**	**3**	**4**	**5**
4. If my feet/ legs become more swollen than usual, I contact my doctor or nurse	**1**	**2**	**3**	**4**	**5**
5. If I gain 2 kilo in one week I contact my doctor or nurse	**1**	**2**	**3**	**4**	**5**
6. I limit the amount of fluids I drink (not more than 1.5 - 2 L/day)	**1**	**2**	**3**	**4**	**5**
7. I take a rest during the day	**1**	**2**	**3**	**4**	**5**
8. If I experience increased fatigue, I contact my doctor or nurse	**1**	**2**	**3**	**4**	**5**
9. I eat a low salt diet	**1**	**2**	**3**	**4**	**5**
10. I take my medication as prescribed	**1**	**2**	**3**	**4**	**5**
11. I get a flu shot every year	**1**	**2**	**3**	**4**	**5**
12. I exercise regularly	**1**	**2**	**3**	**4**	**5**

The European Scale of Self-Care Behavior in Heart Failure (Jaarsma, Strömberg, Martinsson, Drakop, 1999)

PSS

INSTRUCTIONS:

The questions in this scale ask you about your feelings and thoughts during THE LAST MONTH. In each case, please indicate your response by placing an "X" over the circle representing HOW OFTEN you felt or thought a certain way.

	Never 0	Almost Never 1	Sometimes 2	Fairly Often 3	Very Often 4
**1. In the last month, how often have you been upset because of something that happened unexpectedly?**	☐	☐	☐	☐	☐
**2. In the last month, how often have you felt that you were unable to control the important things in your life?**	☐	☐	☐	☐	☐
**3. In the last month, how often have you felt nervous and "stressed"?**	☐	☐	☐	☐	☐
**4. In the last month, how often have you felt confident about your ability to handle your personal problems?**	☐	☐	☐	☐	☐
**5. In the last month, how often have you felt that things were going your way?**	☐	☐	☐	☐	☐
**6. In the last month, how often have you found that you could not cope with all the things that you had to do?**	☐	☐	☐	☐	☐
**7. In the last month, how often have you been able to control irritations in your life?**	☐	☐	☐	☐	☐
**8. In the last month, how often have you felt that you were on top of things?**	☐	☐	☐	☐	☐
**9. In the last month, how often have you been angered because of things that were outside your control?**	☐	☐	☐	☐	☐
**10. In the last month, how often have you felt difficulties were piling up so high that you could not overcome them?**	☐	☐	☐	☐	☐

Save form   Attach to email

SELF-CARE OF HEART FAILURE INDEX

All answers are confidential.

Think about how you have been feeling in the last month or since we last spoke as you complete these items.

SECTION A:

Listed below are common instructions given to persons with heart failure. How routinely do you do the following?

	**Never or rarely**	**Sometimes**	**Frequently**	**Always or daily**
1. Weigh yourself?	1	2	3	4
2. Check your ankles for swelling?	1	2	3	4
3. Try to avoid getting sick (e.g., flu shot, avoid ill people)?	1	2	3	4
4. Do some physical activity?	1	2	3	4
5. Keep doctor or nurse appointments?	1	2	3	4
6. Eat a low salt diet?	1	2	3	4
7. Exercise for 30 minutes?	1	2	3	4
8. Forget to take one of your medicines?	1	2	3	4
9. Ask for low salt items when eating out or visiting others?	1	2	3	4
10. Use a system (pill box, reminders) to help you remember your medicines?	1	2	3	4
