# Supplementary material for: Arabic translation, cross-cultural adaptation, and testing measurement properties of the European heart failure self-care behaviour (EHFScBS-12 and EHFScBS-9)
Source: PLoS One. 2026 Nov 6;21(11):e0361973. doi: 10.1371/journal.pone.0361973 (PMC13742870; doi:10.1371/journal.pone.0361973)
Supplement: Supplementary file 2 [file pone.0361973.s001.docx]

**Forward translation and Cultural Adaptation Procedure:**

| **Original English item** | **A 1 (Translator 1)** | **A 2 (Translator 2)** | **Final selected version** | **Rational for selection** |
| --- | --- | --- | --- | --- |
| 1- I weigh myself every day | أقوم بوزن نفسي كل يوم  (I’m weighing myself everyday) | أزن نفسي كل يوم  (I weigh myself every day) | أزن نفسي كل يوم  (I weigh myself every day) | Closest to original, concise, grammatical correct and commonly used in Arabic. |
| Contact (items 4-5) | أتواصل | اتصل | أتواصل |  |
| Medication | أدويتي  (My medicines) | الدواء  (Medication) | الدواء  (Medication) |  |
| 6- I limit the amount of fluids I drink (not more than 1.5-2 L/day) | أحدد كمية السوائل التي أشربها (لا تزيد عن 1.5-2 لتر يوميًا  (I limit the amount of fluids I drink (not more than 1.5-2 L/day)) | لا تزيد السوائل التي أشربها يومياً عن 1.5 إلى 2 لتر  (I drink no more than 1.5 to 2 liters (of fluids daily | أحدد كمية السوائل التي أشربها (لا تزيد عن 1.5 إلى 2لترباليوم)  (I limit the amount of fluids I drink (not more than 1.5-2 L/day)) |  |
| 2- If I get short of breath، I take it easy | أخلد للراحة  (I take a rest) | أخذ الامر بهدوء  (I take it easy) | أخذ الامر بهدوء  (I take it easy) | Closest to original, and commonly used in Arabic. |

| **Original English wording** | **A 1 (Translator 1)** | **A 2 (Translator 2)** | **Final selected version** | **Rational for selection** |
| --- | --- | --- | --- | --- |
| Failure (Title) | فشل  Failure | قصور  Failure | قصور  Failure | Closest to original, concise, grammatical correct and commonly used in Arabic. |
| I don’t agree at all  (Likert scale) | لا أوافق على الإطلاق | لا أوافق إطلاقاً | لا أوافق إطلاقاً | Closest to original, and commonly used in Arabic. |

**Backward Translation:**

| **The Arabic Translation** | **A 1 (Translator 1)** | **A 2 (Translator 2)** | **Final selected version** | **Rational for selection** |
| --- | --- | --- | --- | --- |
| كيلو | kilo | Kg | Kilo | All were correct but closest to original, concise, grammatical correct and commonly used in Arabic. |
| لقاح | jab | Shot | Shot | Commonly used in Arabic. |
