# Supplementary material for: Arabic translation, cross-cultural adaptation, and testing measurement properties of the European heart failure self-care behaviour (EHFScBS-12 and EHFScBS-9)
Source: PLoS One. 2026 Nov 6;21(11):e0361973. doi: 10.1371/journal.pone.0361973 (PMC13742870; doi:10.1371/journal.pone.0361973)
Supplement: Supplementary file 3 [file pone.0361973.s002.docx]

The European Heart Failure Self-care Behaviour Scale

This scale contains statements about self-care for heart failure. Please answer each statement by circling the number that you think best applies to you. Note that the response options form a scale ranging from "Completely agree" (1) to "Do not agree at all" (5). Even if you are unsure about a particular statement, circle the number that feels most accurate for you.

|  | **I completely agree** |  |  |  | **I don’t agree at all** |
| --- | --- | --- | --- | --- | --- |
| 1. I weigh myself every day | **1** | **2** | **3** | **4** | **5** |
| 2. If I feel short of breath, I take it easy | **1** | **2** | **3** | **4** | **5** |
| 3. If my shortness of breath increases, I contact my doctor or nurse | **1** | **2** | **3** | **4** | **5** |
| 4. If my feet/ legs become more swollen than usual, I contact my doctor or nurse | **1** | **2** | **3** | **4** | **5** |
| 5. If I gain 2 kilo in one week I contact my doctor or nurse | **1** | **2** | **3** | **4** | **5** |
| 6. I limit the amount of fluids I drink (not more than 1.5 - 2 L/day) | **1** | **2** | **3** | **4** | **5** |
| 7. I take a rest during the day | **1** | **2** | **3** | **4** | **5** |
| 8. If I experience increased fatigue, I contact my doctor or nurse | **1** | **2** | **3** | **4** | **5** |
| 9. I eat a low salt diet | **1** | **2** | **3** | **4** | **5** |
| 10. I take my medication as prescribed | **1** | **2** | **3** | **4** | **5** |
| 11. I get a flu shot every year | **1** | **2** | **3** | **4** | **5** |
| 12. I exercise regularly | **1** | **2** | **3** | **4** | **5** |

The European Scale of Self-Care Behavior in Heart Failure (Jaarsma, Strömberg, Martinsson, Drakop, 1999)

The European Heart Failure Self-care Behaviour Scale

This scale contains statements about self-care for heart failure. Please answer each statement by circling the number that you think best applies to you. Note that the response options form a scale ranging from "Completely agree" (1) to "Do not agree at all" (5). Even if you are unsure about a particular statement, circle the number that feels most accurate for you.

|  | **I completely agree** |  |  |  | **I don’t agree at all** |
| --- | --- | --- | --- | --- | --- |
| 1. I weigh myself every day | **1** | **2** | **3** | **4** | **5** |
| 2. If my shortness of breath increases, I contact my doctor or nurse | **1** | **2** | **3** | **4** | **5** |
| 3. If my feet/ legs become more swollen than usual, I contact my doctor or nurse | **1** | **2** | **3** | **4** | **5** |
| 4. If I gain 2 kilo in one week I contact my doctor or nurse | **1** | **2** | **3** | **4** | **5** |
| 5. I limit the amount of fluids I drink (not more than 1.5 - 2 L/day) | **1** | **2** | **3** | **4** | **5** |
| 6. If I experience increased fatigue, I contact my doctor or nurse | **1** | **2** | **3** | **4** | **5** |
| 7. I eat a low salt diet | **1** | **2** | **3** | **4** | **5** |
| 8. I take my medication as prescribed | **1** | **2** | **3** | **4** | **5** |
| 9. I exercise regularly | **1** | **2** | **3** | **4** | **5** |

The European Scale of Self-Care Behavior in Heart Failure (Jaarsma, Strömberg, Martinsson, Drakop, 1999)
